# Double arterial cannulation versus right axillary artery cannulation for acute type A aortic dissection: a retrospective study

**DOI:** 10.1186/s13019-021-01714-5

**Published:** 2021-11-07

**Authors:** He Zhang, Wei Xie, Yuzhou Lu, Tuo Pan, Qing Zhou, Yunxing Xue, Dongjin Wang

**Affiliations:** 1 Department of Cardiothoracic Surgery, Nanjing Drum Tower Hospital, Peking Union Medical College, Chinese Academy of Medical Sciences, Graduate School of Peking Union Medical College, Nanjing, 210008 Jiangsu China; 2 Department of Cardiothoracic Surgery, Nanjing Drum Tower Hospital, The Affiliated Hospital of Nanjing University Medical School, Number 321 Zhongshan Road, Nanjing, 210008 Jiangsu China

**Keywords:** Aortic dissection, Cannulation, Malperfusion

## Abstract

**Background:**

Cannulation strategy in surgery for acute type A aortic dissection (ATAAD) remains controversial. We aimed to retrospectively analyze the safety and efficacy of double arterial cannulation (DAC) compared with right axillary cannulation (RAC) for ATAAD.

**Methods:**

From January 2016 to December 2018, 431 ATAAD patients were enrolled in the study. Patients were divided into DAC group (n = 341) and RAC group (n = 90). Propensity score matching analysis was performed to compare the early and mid-term outcomes between these two groups. To confirm the organ protection effect by DAC, intraoperative blood gas results and cardiopulmonary bypass parameters were compared between the two groups.

**Results:**

Demographics and preoperative comorbidities were comparable between two groups, while patients in DAC group were younger than RAC group (51.55 ± 13.21 vs. 56.07 ± 12.16 years, *P* < 0.001). DAC had a higher incidence of limb malperfusion (18.2% vs. 10.0%, *P* = 0.063) and lower incidence of coronary malperfusion (5.3% vs. 12.2%, *P* = 0.019). No significant difference in cardiopulmonary bypass and cross-clamp time was found between the two groups. The in-hospital mortality was 13.5% (58/431), while there was no difference between the two groups (13.5% vs. 13.3%; *P* = 0.969). Patients who underwent DAC had higher incidence of postoperative stroke (5.9% vs. 0%, *P* = 0.019) and lower incidence of postoperative acute kidney injury (AKI) (24.7% vs. 40.3%; *P* = 0.015). During a mean follow-up period of 31.8 (interquartile range, 25–45) months, the overall survival was 81.5% for DAC group and 78.0% for RAC group (*P* = 0.560). Intraoperative blood gas results and cardiopulmonary bypass parameters showed that DAC group had more intraoperative urine output volume than RAC group (*P* = 0.05), and the time of cooling (*P* = 0.04) and rewarming (*P* = 0.04) were shorter in DAC group.

**Conclusions:**

DAC will not increase the surgical risks compared to RAC, but could reduce the incidence of postoperative AKI which may be benefit for renal protection.

**Supplementary Information:**

The online version contains supplementary material available at 10.1186/s13019-021-01714-5.

## Introduction

Acute Type A aortic dissection (ATAAD) is a complex emergency cardiovascular disease with high mortality and morbidity rates. Management of ATAAD is still a challenge for cardiothoracic surgeons. According to the results of IRAD, the in-hospital mortality rates had fallen from 31 to 22%, the operative mortality ranged from 25 to 18% [1]. It is important to establish fast and safe cardiopulmonary bypass (CPB) to ensure adequate systemic perfusion for ATAAD surgery, however, there is still no standard of cannulation strategy. Femoral artery and axillary artery are the most common cannulation approaches for ATAAD. Femoral arterial cannulation can be constructed immediately in patients with unstable hemodynamic conditions but will increase the incidence of stroke due to retrograde perfusion [2]. Right axillary cannulation (RAC) can provide antegrade perfusion which may be benefit for brain protection but may cause end-organ malperfusion because of limited flow rate [3, 4]. To improve the perfusion in ATAAD surgery, double arterial cannulation (DAC) combined right axillary artery with femoral artery was used and was benefit for patients with preoperative malperfusion [5, 6]. Furthermore, the studies of DAC in ATAAD were limited and lacked follow-up results, which were difficult to state whether ATAAD patients can benefit from DAC.

The technique of double arterial cannulation has been the standard cannulation strategy for ATAAD at our center since 2016. We aimed to study the safety and effectiveness of DAC through retrospectively comparing with RAC.

## Methods

### Patients and study design

**Fig. 1:**
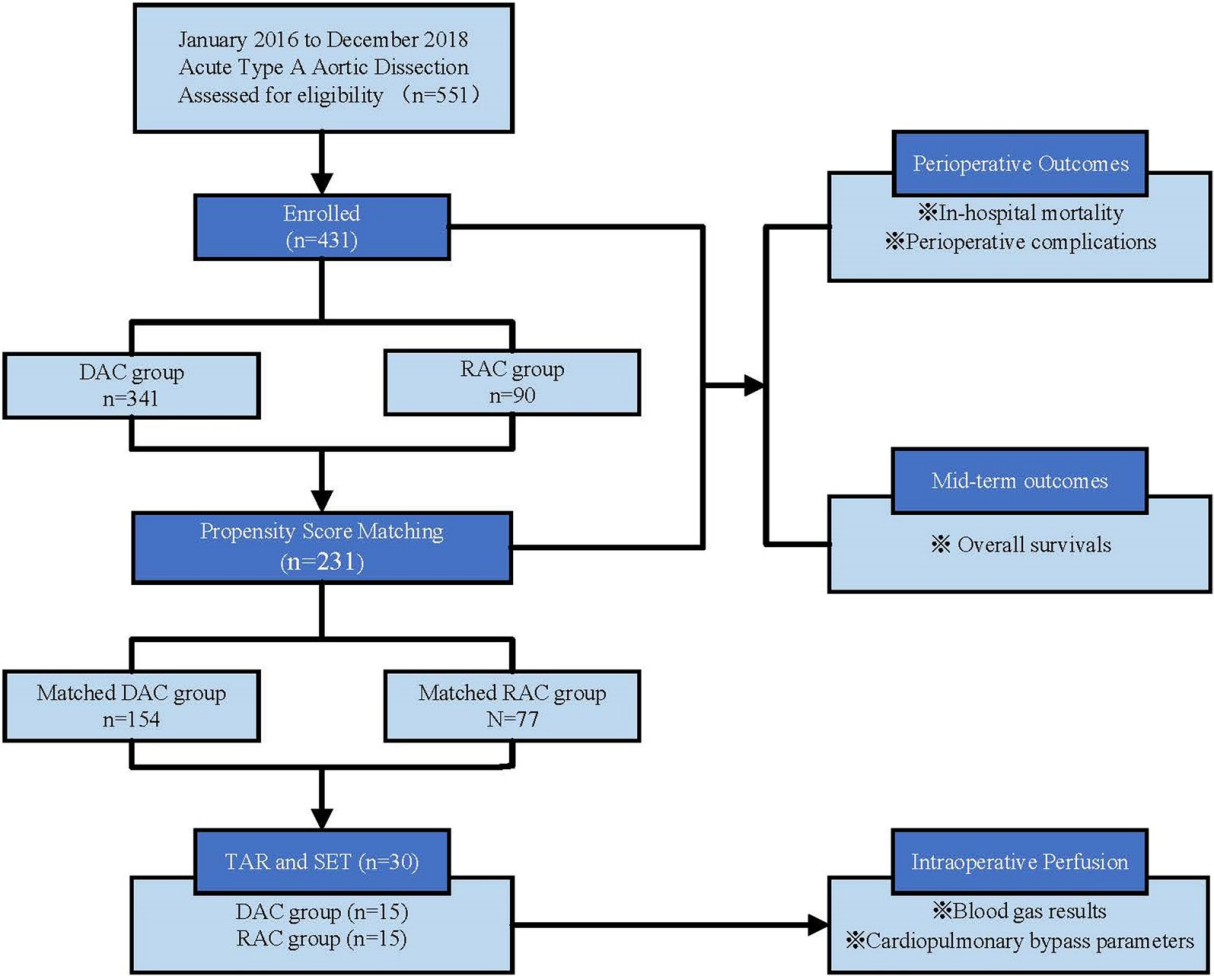
The flow chart of retrospective study design

From January 2016 to December 2018, 551 patients were diagnosed with ATAAD by computerized tomography angiogram (CTA) and underwent emergency operation in Cardiothoracic Surgery of Nanjing Drum Tower Hospital. Among them, 341 patients were cannulated with DAC and 90 patients with RAC. We compared demographic information, preoperative clinical characteristics, operation strategies and early and mid-term outcomes between two groups, we matched the patients on the basis of propensity scores to make comparable clinical characteristics. Furthermore, we compared the intraoperative perfusion status in patients without preoperative malperfusion syndrome who underwent total arch replacement (TAR) and stented elephant trunk (SET) between the two groups through blood gas analysis and cardiopulmonary bypass parameters. The flow chart of retrospective study design is shown in Fig. 1.

The study was approved by ethics committee of Nanjing Drum Tower Hospital (NO.2020-185-01). The requirement to obtain informed consent from patients was waived, and all authors had full control of the data and information of this study.

### Surgical approach

All operations were performed through intravenous and inhaled anesthesia, endotracheal intubation, upper and lower extremity arterial puncture monitoring blood pressure and placing an esophageal ultrasound probe. Patients in RAC group were cannulated before median sternotomy. Right axillary artery cannulation can be established directly or indirectly. A 3–5 cm incision was made 1 cm below the right clavicle, paralleling to the long axis of the clavicle. The right axillary artery was then identified by palpation and mobilised avoiding the brachial plexus posteriorly. After the full dose of heparin required for CPB was administered, proximal and distal end of axillary artery were blocked. The axillary artery can be assessed by cutdown and direct cannulation by 18F-24F graft or placed in an 8-mm Dacron graft that is anastomosed to the axillary artery in an end-to-side manner. Patients in DAC group take the same protocol of axillary artery cannulation, femoral artery can be assessed by cutdown and direct cannulation by 22F-24F graft.

Routine retrograde myocardial perfusion was used through coronary sinus. When the bladder temperature (core temperature) dropped to 18–22 °C, we arrested the systemic circulation and perfused the cerebral with selected cerebral perfusion (SCP) or Retrograde cerebral perfusion (RCP). Details of proximal repair and distal repair have been described previously. Generally, in the patients with arch dilation (≥ 45 mm) and intima tears in the arch and damaged structure of arch, we chose the total arch replacement, stented elephant trunk was applied the same time if replacing the total arch. Otherwise, partial aortic arch replacement or antegrade-implantation arch stent were used [7, 8]. Core temperature begun to recover after distal repair anastomosis finished. During the rewarm phase, we repaired the aortic root. The root reinforcement reconstruction method was as reported before [9, 10]. For patients with indications for root replacement, Bentall procedure or David procedure were selected.

### Follow-up

Patients were encouraged to receive CTA and transesophageal echocardiography (TEE) in the following 1, 3, 6 and 12 months and annually after surgery. The data were recorded at the outpatient visit or by phone call interview. We recorded the all-cause mortality as the follow-up endpoint.

### Definitions

ATAAD was defined less than two weeks from symptom onset. Organ malperfusion was diagnosed by the description of Pacini [11]. Shock was defined as systolic blood pressure below 90 mmHg regardless of vasoactive agent usage. The extent of tamponade was diagnosed by CT or echocardiography. The RIFLE criteria were used to define AKI in patients with an increase in either sCr values of > 50% or > 27 µmol/l or an eGFR decrease of > 25% within the first 48 post-operative hours compared with baseline [12].

### Statistical analysis

SPSS 25.0 software was used for data processing and analysis. For the measurement data, if it conforms to the normal distribution, it was expressed as mean ± standard deviation (mean ± SD). The counting data was expressed in the ratio of rate or composition (n, %). *T*-test was used for the measurement data conforming to normal distribution and homogeneity of variance, and rank combination test was used for the measurement data not conforming to normal distribution or homogeneity of variance. Chi square test or Fisher exact probability method were used for counting data. *P* value less than 0.05 was considered to have statistical difference.

We acknowledge the existence of bias in our study, to achieve a sound scientific conclusion, we used the propensity score match to adjust for an indication. Propensity score 1-to-2 matching was utilized with the nearest neighbour algorithm without replacement and a 0.05 caliper setting. Sex, Age, BMI, Diabetes, Stroke history, COPD, CHD, ESKD, Marfan syndrome, Cardiac surgery, Smoking, Alcohol, Shock, Tamponade and Preoperative malperfusion were put into a logistic regression model to estimate the propensity score. Absolute standardized differences were used to assess pre-match imbalances and post-match balance in baseline covariates. The best balance is reflected by a standardized difference below 10%. A standardized mean difference plot indicating the bias reduction after matching is shown in Additional file 1: Fig. E1.

## Results

The demographics and preoperative clinical presentations for the DAC and RAC groups are listed in Table 1. The mean age for all patients were 52.49 ± 13.10 years, the patients in DAC group were younger than RAC group (*P* < 0.001). Most patients were male (320/431, 74.2%) and had history of hypertension with irregular medication (322/431, 74.7%).

**Table 1:** Preoperative characteristics for the DAC and RAC groups

Variables	DAC group (n = 341)	RAC group (n = 90)	*P* value	Matched DAC group (n = 154)	Matched RAC group (n = 77)	*P* value
Sexy(male)	248 (72.7%)	72 (80.0%)	0.160	126 (81.8%)	63 (81.8%)	1.000
Age	51.55 ± 13.21	56.07 ± 12.16	< 0.001	54.48 ± 12.48	55.04 ± 11.95	0.745
BMI	25.75 ± 4.06	25.39 ± 4.19	0.462	25.33 ± 3.61	25.13 ± 4.19	0.705
Hypertension	250 (73.3%)	72 (80.0%)	0.194	119 (77.3%)	63 (81.8%)	0.426
Diabetes	13 (3.8%)	3 (3.3%)	0.831	6 (3.9%)	2 (2.6%)	0.611
Stroke history	8 (2.3%)	3 (3.3%)	0.597	4 (2.6%)	1 (1.3%)	0.523
COPD	4 (1.2%)	1 (1.1%)	0.961	3 (1.9%)	1 (1.3%)	0.721
CHD	8 (2.3%)	1 (1.1%)	0.466	3 (1.9%)	1 (1.3%)	0.721
ESKD	9 (2.6%)	1 (1.1%)	0.392	0	1 (1.3%)	0.156
Marfan	5 (1.5%)	2 (2.2%)	0.614	2 (1.3%)	2 (2.6%)	0.476
Cardiac surgery	16 (4.7%)	0	0.036	0	0	1.000
Smoking	69 (20.2%)	17 (18.9%)	0.776	32 (20.8%)	14 (18.2%)	0.641
Alcohol	54 (15.8%)	14 (15.6%)	0.948	24 (15.6%)	9 (11.7%)	0.425
Shock	8 (2.3%)	3 (3.3%)	0.597	3 (1.9%)	1 (1.3%)	0.721
Tamponade	24 (7.0%)	6 (6.7%)	0.902	11 (7.1%)	4 (5.2%)	0.571
Malperfusion						
Brain	30 (8.8%)	8 (8.9%)	0.978	8 (5.2%)	8 (10.4%)	0.143
Coronary	18 (5.3%)	11 (12.2%)	0.019	8 (5.2%)	5 (6.5%)	0.686
Limb	62 (18.2%)	9 (10.0%)	0.063	15 (9.7%)	9 (11.7%)	0.647
Visceral	21 (6.2%)	11 (12.2%)	0.051	13 (8.4%)	9 (11.7%)	0.428

*BMI* Body mass index; *COPD* chronic obstructive pulmonary disease; *CHD* coronary heart disease; *ESKD* end stage kidney disease

Sixteen patients had cardiovascular operations and were all in DAC group. Thirty patients (6 in RAC group and 24 in DAC) presenting with cardiac tamponade were received emergency surgery directly from emergency room to operative room. Limb malperfusion was the most common symptom in this study, which was more common in DAC group than RAC group (18.2% vs. 10%; *P* = 0.063). Coronary artery malperfusion (12.2% vs. 5.3%; *P* = 0.019) and visceral malperfusion (12.2% vs. 6.2%; *P* = 0.051) had presented more obviously in RAC group than DAC group. Brain malperfusion was similar between two groups (8.9% vs. 8.8%; *P* = 0.978). After propensity score matching, we identified two matched groups (154 patients in DAC group and 77 patients in RAC group) with similar baseline characteristics which were shown in Table 1. A standardized mean difference plot indicating the bias reduction after matching is listed in Additional file 1: Fig. E1.

Surgical details are shown in Table 2. The mean time of cardiopulmonary bypass, cross-clamp and DHCA were 251.24 ± 79.81, 174.63 ± 56.85, 30.04 ± 10.77 min. DAC group presented longer bypass time, cross-clamp time and DHCA time, but no statistical difference between the two groups. Proximal reconstruction procedures were similar between two groups, Root reconstruction procedures were most common in root surgery (70.9%, 306/431), Bentall procedure came second (24.6%, 106/431). Total arch replacement and arch stent procedures were performed in a high percent of both two groups.

**Table 2:** Operative details for the DAC and RAC groups

Variables	DAC group (n = 341)	RAC group (n = 90)	*P* value	Matched DAC group (n = 154)	Matched RAC group (n = 77)	*P* value
Site of tears			0.286			0.547
Ascending aorta	191 (56.0%)	42 (46.7%)		79 (51.3%)	37 (48.1%)	
Aortic arch	98 (28.7%)	28 (31.1%)		45 (29.2%)	21 (27.3%)	
Descending aorta	22 (6.5%)	10 (11.1%)		11 (7.1%)	10 (13.0%)	
Unseen	30 (8.8%)	10 (11.1%)		19 (12.3%)	9 (11.7%)	
Root Surgery			0.168			0.181
Untreated	9 (2.6%)	1 (1.1%)		4 (2.6%)	0	
Root reconstruction	237 (69.5%)	69 (76.7%)		102 (66.2%)	59 (76.6%)	
Bentall	90 (26.4%)	16 (17.8%)		46 (29.9%)	16 (20.8%)	
Wheat	1 (0.3%)	1 (1.1%)		0	0	
David	4 (1.2%)	3 (3.3%)		2 (1.3%)	2 (2.6%)	
Arch Surgery			0.719			0.969
Untreated	10 (2.9%)	3 (3.3%)		3 (1.9%)	1 (1.3%)	
Hemi-arch	44 (12.9%)	9 (10.0%)		20 (13.0%)	9 (11.7%)	
Total-arch	181 (53.1%)	45 (50.0%)		78 (50.6%)	39 (50.6%)	
Arch stent	106 (31.1%)	33 (36.7%)		53 (34.4%)	28 (36.4%)	
Descending stent	289 (84.8%)	79 (87.8%)	0.470	130 (84.4%)	67 (87.0%)	0.599
Perfusion Method			0.859			0.616
Without perfusion	1 (0.3%)	0		0	0	
ACP	336 (98.8%)	89 (98.9%)		153 (99.4%)	76 (98.7%)	
RCP	3 (0.9%)	1 (1.1%)		1 (0.6%)	1 (1.3%)	
Operation time (h)	8.18 ± 1.93	8.52 ± 1.87	0.130	8.09 ± 1.96	8.50 ± 1.85	0.127
CPB (min)	251.92 ± 82.42	248.66 ± 1.87	0.734	241.68 ± 72.41	244.30 ± 70.50	0.795
Crossclamp time(min)	176.12 ± 57.10	168.93 ± 56.17	0.292	168.59 ± 55.19	166.71 ± 56.78	0.810
DHCA (min)	30.51 ± 11.19	28.21 ± 8.87	0.077	28.87 ± 9.93	28.09 ± 8.92	0.568

*ACP* Anterograde cerebral perfusion; *RCP* retrograde cerebral perfusion; *CPB* cardiopulmonary bypass; *DHCA* deep hypothermic circulatory arrest

Overall in-hospital mortality was 13.5% (58/431), there were no significant differences between two groups, which was 13.3% in RAC group and 13.5% in DAC group (*P* = 0.969). Postoperative complications differ significantly between the two groups: the duration of mechanical ventilation was shorter in DAC group than RAC group (*P* = 0.026); the incidence of stroke (*P* = 0.019) and surgical site infection (*P* = 0.043) was higher in DAC group than RAC group. Moreover, the incidence of AKI was much higher in RAC group than DAC group (37.8% vs. 26.7%, *P* = 0.039), while the usage of continuous renal replacement therapy (CRRT) had no significantly difference between groups (*P* = 0.969). Other postoperative complications did not differ significantly between groups. After propensity score matching, only the incidence of AKI was still much lower in DAC group than RAC group (24.7% vs. 40.3%, *P* = 0.015). Perioperative results before and after propensity score matching were shown in Table 3.

**Table 3:** Postoperative results for the DAC and RAC groups

Variables	DAC group (n = 341)	RAC group (n = 90)	*P* value	Matched DAC group (n = 154)	Matched RAC group (n = 77)	*P* value
Mortality	46 (13.5%)	12 (13.3%)	0.969	26 (16.9%)	9 (11.7%)	0.299
Mechanical Ventilation (h)	42.39 ± 46.03	56.43 ± 57.01	0.026	41.28 ± 42.06	51.69 ± 53.95	0.165
Re-intubation	23 (6.7%)	3 (3.3%)	0.227	13 (8.4%)	3 (3.9%)	0.200
Tracheotomy	8 (2.3%)	5 (5.6%)	0.113	4 (2.6%)	4 (5.2%)	0.309
Re-exploration	21 (6.2%)	4 (4.4%)	0.536	5 (3.2%)	3 (3.9%)	0.799
Stroke	20 (5.9%)	0	0.019	6 (3.9%)	0	0.079
Paraplegia	5 (1.5%)	0	0.248	1 (0.6%)	0	0.479
AKI	91 (26.7%)	34 (37.8%)	0.039	38 (24.7%)	31 (40.3%)	0.015
CRRT	46 (13.5%)	12 (13.3%)	0.969	20 (13.0%)	9 (11.7%)	0.779
SSI	15 (4.4%)	0	0.043	7 (4.5%)	0	0.057
Limb malperfusion	6 (1.8%)	1 (1.1%)	0.665	2 (1.3%)	1 (1.3%)	1.000
Visceral malperfusion	8 (2.3%)	1 (1.1%)	0.466	7 (4.5%)	1 (1.3%)	0.203
ICU (day)	6.74 ± 8.27	6.82 ± 6.70	0.935	6.28 ± 7.33	6.45 ± 5.25	0.854
In-hospital time (day)	22.42 ± 13.59	23.19 ± 14.31	0.637	21.18 ± 12.41	23.59 ± 14.44	0.194

*AKI* Acute kidney injury; *CRRT* continuous renal replacement therapy; *SSI* surgical site infection; *ICU* intensive care unit

**Fig. 2:**
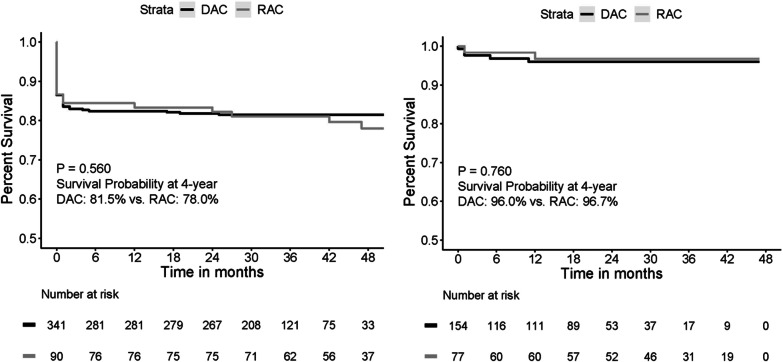
Kaplan–Meier curves for overall survival. Shown is the overall survival in the DAC and RAC groups, as well as two matched groups during follow-up

Follow-up was completed in 95.4% of patients (411/431), with a mean follow-up time of 31.8 months (interquartile range [IQR], 25–45 months). The overall survival was 81.5% in DAC group and 78.0% in RAC group which was similar between two groups (*P* = 0.560). The overall survival is demonstrated in the Fig. 2.

The information of 30 ATAAD patients who underwent TAR and SET was listed in Additional file 2: Table E1. The results of intraoperative blood gas analysis were no difference between two groups. Cardiopulmonary bypass parameters showed that the cooling time and rewarming time were shorter in DAC group than RAC group (*P* = 0.04). Moreover, DAC group had more average intraoperative urine volume than RAC group (*P* = 0.05). The details of blood gas analysis and cardiopulmonary bypass are shown in Table 4.

**Table 4:** Blood gas analysis and cardiopulmonary bypass records of patients who underwent TAR and SET

	RAC group (n = 15)	DAC group (n = 15)	*P* value
BSA (m^2^)	1.87 ± 0.21	1.89 ± 0.17	0.79
Preoperative urine volume (ml)	222.7 ± 184.8	237.3 ± 113.5	0.80
Intraoperative urine volume (ml)	403.3 ± 345.1	676 ± 389.7	0.05
Lowest HCT	20.97 ± 5.82	23.39 ± 3.56	0.18
Highest HCT	27.54 ± 3.28	29.99 ± 5.55	0.15
Last HCT	27.03 ± 3.50	28.33 ± 5.78	0.46
Highest pCO_2_	46.73 ± 10.9	48.19 ± 4.86	0.59
Last pCO_2_	35.73 ± 4.70	44.15 ± 5.13	< 0.01
Lowest COP	279.9 ± 5.48	276.5 ± 15.44	0.44
Highest COP	293.9 ± 7.90	298.7 ± 9.73	0.17
Last COP	292.6 ± 8.79	298.7 ± 9.73	0.10
Highest lactic acid	5.49 ± 2.40	6.54 ± 3.64	0.34
Last lactic acid	4.89 ± 2.40	6.25 ± 3.75	0.25
Highest volume (ml/min)	5047 ± 467.3	5087 ± 297.3	0.78
Lowest volume (ml/min)	2653 ± 819.3	2660 ± 489.6	0.98
Lowest pumping pressure	131.4 ± 27.03	136.1 ± 24.1	0.62
Highest pumping pressure	260.8 ± 34.99	238.9 ± 35.53	0.09
Cooling time (min)	45.2 ± 6.61	39.67 ± 7.64	0.04
Rewarming time (time)	98.6 ± 29.6	80.5 ± 14.5	0.04
Percent of axillary MAP < 70 mmHg	0.60 ± 0.17	0.65 ± 0.13	0.35
Percent of axillary MAP < 60 mmHg	0.22 ± 0.18	0.29 ± 0.21	0.32
Percent of femoral MAP < 70 mmHg	0.63 ± 0.17	0.59 ± 0.14	0.52
Percent of femoral MAP < 60 mmHg	0.27 ± 0.18	0.23 ± 0.19	0.50

*BSA* Body surface area; *HCT* hematocrit; *pCO2* partial pressure of carbon dioxide; *COP* colloid osmotic pressure; *MAP* mean arterial pressure

## Discussion

Establishing an efficient and safe cardiopulmonary bypass to maintain adequate systemic perfusion is essential in ATAAD surgery. The optimal cannulation site should meet the physiology needs of important organs in the whole body, provide a clean surgical field and reduce the complications associated with cannulation. According to a survey across European cardiac centers [13]: In both acute and chronic settings, the right subclavian–axillary approach is the favourite site for cannulation (54% and 48%, respectively). The second favoured choice differs depending on the clinical presentation: for acute conditions, the femoral approach is preferred, while, for chronic conditions, the ascending aorta is preferred, both accounting for 28% of the cases. Femoral artery cannulation can be used quickly and provide enough perfusion flow for whole body, therefore, it is preferred in hemodynamic unstable patients instead of axillary artery cannulation which may occasionally be too time-consuming [14, 15]. However, femoral artery cannulation hast a high rate of false lumen perfusion, limb malperfusion and cerebral complications a due to a retrograde flow [16]. In our center, femoral artery cannulation was used mostly in the unstable haemodynamics patients (97/551, 17.6%). As an alternative cannulation strategy, axillary artery cannulation has been advocated by many surgeons [17]. The collateral circulation of neck and shoulder is rich, and the distal limbs are not easy to be ischemic necrosis and involved by atherosclerosis and dissection, which can effectively avoid retrograde embolization, avoid the expansion of dissection range and facilitate antegrade selective cerebral perfusion [14, 18]. Several researches had shown that RAC can reduce intraoperative and postoperative mortality, lower rates of cerebral complications and malperfusion and fewer reoperation rates which could improve both short-term and long-term outcomes of ATAAD compared with central aortic or femoral artery cannulation [19–21]. However, the diameter of the axillary artery is smaller compared to femoral artery, single axillary artery cannulation may lead to end-organ malperfusion in some ATAAD patients.

Double arterial (axillary artery combined with femoral artery) cannulation has reliable circulatory support which provides anterograde and retrograde blood flow at the same time to achieve the best systemic perfusion strategy, results of DAC strategy are scarcely published with limited cases available. Minatoya [5] reported a group of 88 AAD patients used DAC, the results showed that DAC approach was associated with a low mortality even in AAD patients with malperfusion. Recently, Lin et al. [21] reported that ATAAD patients who underwent DAC had lower in-hospital mortality and lower incidence of malperfusion-related complications than those who underwent single arterial cannulation, three-year cumulative survival was also better in DAC group. However, we didn’t find any difference on in-hospital mortality and follow-up survival between DAC and RAC group in our 431 patients’ study. Our results were similar to the study from Kusadokoro et al. [22], they found that DAC had acceptable early and long-term follow-up results for both planned and unplanned (rescue) ATAAD surgery.

It arouses curiosity whether cannulation strategies differ in terms of perioperative parameters in ATAAD patients. Kusadokoro et al. [22] reported that they preferred DAC in patients with true lumen stenosis and perioperative leg malperfusion. However, even with propensity score matching, the trend that DAC group were younger and with lower incidence of perioperative shock was still seen. This trend was similar to our cannulation strategy, we preferred the DAC strategy in younger patients with perioperative limb and visceral malperfusion. We consider that the axillary artery combined with femoral artery may improve true lumen narrowing and perfusion status which may be benefit for organ protection.

Cerebrovascular injury is one of the major causes of morbidity and mortality of ATAAD surgery and cannulation with femoral artery was recognized as a risk factor for inferior outcome [23, 24]. In our study, we found that the incidence of postoperative stroke was higher in DAC group than RAC group due to addition of femoral artery. While the incidence of postoperative stroke in DAC group (5.9%) and RAC group (0%) were much lower than reports of German Registry for Acute Aortic Dissection Type A (GERAADA) [25]. The lower incidence of postoperative cerebrovascular injury in both DAC and RAC might be associated with adequate cerebral perfusion and cerebral monitoring using the bilateral cerebral oxygen.

AKI is another important early complication following ATAAD-repair that increases patients’ mortality, which was reported ranging from 40 to 55% [26]. In our study, the incidence of AKI was 29.0% in all patients which was lower than what reported in literature. Moreover, the incidence of AKI was much lower in DAC group than RAC (26.7% vs. 37.8%, *P* = 0.039). Similar to previous studies that age, obesity, hypertension and prolonged CPB times were independent risk factors for postoperative AKI [27, 28], we found that patients in DAC group were younger than RAC group. In addition, the duration of mechanical ventilation was shorter in DAC group (42.39 ± 46.03 h) than RAC group (56.43 ± 57.01 h) which demonstrated our previous findings. Wang Z and his colleagues in our center reported that shorten mechanical ventilation duration as much as possible might help reducing postoperative AKI incidence [29]. The relationship between prolonged mechanical ventilation and postoperative AKI may be explained by three possible mechanisms: (I) through effects on arterial blood gases; (II) through an effect on systemic and renal blood flow; (III) by triggering a pulmonary inflammatory reaction induced during biotrauma that further mediates systemic changes [30]. We made propensity score matching (PSM) to avoid the bias of age and preoperative conditions, the incidence of AKI was still much lower in DAC group than RAC group after PSM (24.7% vs. 40.3%, *P* = 0.015). To confirm what we found, we compared intraoperative blood gas results and cardiopulmonary bypass parameters in patients who underwent total arch replacement (TAR) and stented elephant trunk (SET). In this cohort, patients in DAC group had more intraoperative urine volume than RAC group (*P* = 0.05) which may indicate that DAC strategy could provide better kidney perfusion during ATAAD surgery. Furthermore, the cooling time and rewarming time were all shorter in DAC group than RAC group, this may be explained that DAC could introduce both forward perfusion and reverse perfusion to achieve the best systemic perfusion effect, and the two-way blood flow has better systemic perfusion stability. Based on what we found in this study, we thought that DAC could reduce the incidence of AKI by providing better kidney perfusion.

## Conclusions

Double arterial cannulation is safe for acute Type A aortic dissection operation, which has similar perioperative and mid-term outcomes compared to axillary arterial cannulation. Using DAC to establish CPB could reduce the incidence of postoperative acute kidney injury without increasing other surgical risks, which may have better renal protection.

## Limitations

This study is a retrospective and non-randomized single center design. Therefore, bias of selecting patients might have influenced the homogeneity of the groups, including the age and malperfusions before operation. Moreover, limited follow-up period among 12 months to 59 months.

## Supplementary Information

**Additional file 1: Figure E1.** Absolute Standardized Mean Difference.

**Additional file 2: Table E1.** Demographic characteristics and operative information for patients who underwent TAR and SET.

## Data Availability

The datasets generated and analyzed during the current study are available from the corresponding author on reasonable request.

## Abbreviations

ATAAD: Acute type A aortic dissection
DAC: Double arterial cannulation
RAC: Right axillary cannulation
AKI: Acute kidney injury
CPB: Cardiopulmonary bypass
CTA: Computed tomography angiography
TAR: Total arch replacement
SET: Stented elephant trunk
SCP: Selected cerebral perfusion
CABG: Coronary artery bypass grafting
BMI: Body mass index
COPD: Chronic obstructive pulmonary disease
ESKD: End stage kidney disease
CRRT: Continuous renal replacement therapy
ICU: Intensive care unite
