# Supplementary figures and images for: Double arterial cannulation versus right axillary artery cannulation for acute type A aortic dissection: a retrospective study
Source: J Cardiothorac Surg. 2021 Nov 7;16:326. doi: 10.1186/s13019-021-01714-5 (PMC8574002; doi:10.1186/s13019-021-01714-5)

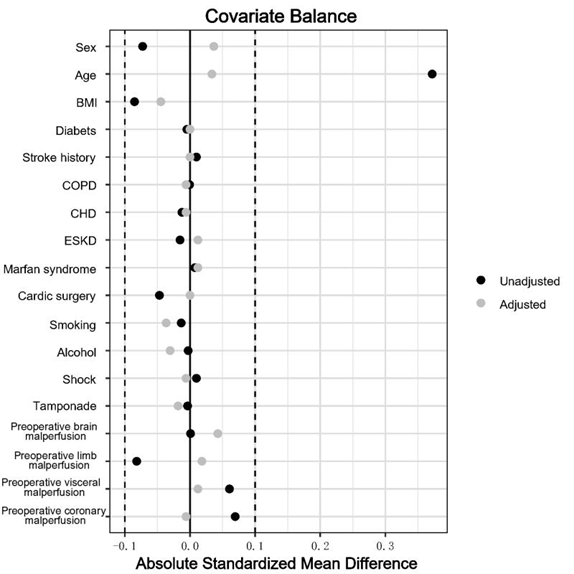

Supplement: Supplementary file 1 — Additional file 1: Figure E1. Absolute Standardized Mean Difference. [file 13019_2021_1714_MOESM1_ESM.tif]
