# Supplementary material for: Double arterial cannulation versus right axillary artery cannulation for acute type A aortic dissection: a retrospective study
Source: J Cardiothorac Surg. 2021 Nov 7;16:326. doi: 10.1186/s13019-021-01714-5 (PMC8574002; doi:10.1186/s13019-021-01714-5)
Supplement: Supplementary file 2 — Additional file 2: Table E1. Demographic characteristics and operative information for patients who underwent TAR and SET. [file 13019_2021_1714_MOESM2_ESM.docx]

**Table E1. Demographic characteristics and operative information for patients who underwent TAR and SET**

| Variables | DAC group  (n=15) | RAC group  (n=15) | P value |
| --- | --- | --- | --- |
| Sexy (male) | 13(86.7%) | 12(80.0%) | ＞0.99 |
| Age | 48.3±12.3 | 50.3±16.3 | 0.71 |
| BMI | 24.4±3.3 | 25.5±4.2 | 0.49 |
| Emergency Surgery | 15(100.0%) | 15(100.0%) | 1.00 |
| CPB (min) | 225.8±24.7 | 206.5±40.7 | 0.128 |

*BMI,* Body Mass Index; *CPB,* Cardiopulmonary bypass
